# Saving the split: protocol for an umbrella review on therapeutic approaches for cracked tooth syndrome

**DOI:** 10.1186/s13643-025-03048-y

**Published:** 2026-01-07

**Authors:** Supreet Kaur, Lakshmi Puzhankara, Neetha Shenoy, Sandya Kini K, Deepak Kumar Singhal

**Affiliations:** 1 Department of Conservative Dentistry & Endodontics, Manipal College of Dental Sciences, Manipal Academy of Higher Education, Manipal, Karnataka 576104 India; 2 Department of Periodontology, Manipal College Of Dental Sciences, Manipal Academy of Higher Education, Manipal, Karnataka 576104 India; 3 Department of Public Health Dentistry, Manipal College Of Dental Sciences, Manipal Academy of Higher Education, Manipal, Karnataka 576104 India

**Keywords:** Cracked tooth syndrome, Therapeutic modalities, Umbrella review, Systematic review, Meta-analysis, Tooth fracture management, Endodontic treatment, Restorative dentistry, Evidence-based dentistry, Umbrella review protocol

## Abstract

**Background:**

Cracked tooth syndrome (CTS) is challenging to diagnose and manage due to its variable clinical presentation, resulting in inconsistent conclusions across existing reviews. Although several systematic reviews have investigated interventions independently, the evidence remains disparate, highlighting the need for an umbrella review to consolidate the different pieces of evidence and address any inconsistencies, thereby informing clinical recommendations.

**Methods:**

This umbrella review will include published systematic reviews and meta-analyses focusing on treatment for CTS. Various databases like PubMed, Cochrane Library, Scopus, Embase, and Web of Science will be searched till June 01, 2025. Two reviewers will independently perform the article screening, study identification, data extraction, and risk of bias assessment using the AMSTAR-2 tool. Overlap between relevant primary sources will be evaluated with the Corrected Covered Area (CCA) method. Certainty of evidence will be determined using the GRADE approach. The results will be reported as a narrative synthesis accompanied by summary tables. There will be an inclusion of only English-language publications, and the possibility of language bias will be recognized.

**Discussion:**

This umbrella review will offer a broad and detailed summary of the available evidence pertaining to the management of CTS to facilitate the implementation of more uniform, evidence-based clinical decisions. The review, through assessing the quality of the methods used, locating the points of agreement or dispute, and indicating the gaps in the research, will be a guide for both the present practice and the next research directions.

**Systematic review registration:**

PROSPERO CRD420250648720.

**Supplementary Information:**

The online version contains supplementary material available at 10.1186/s13643-025-03048-y.

## Background

Cracked Tooth Syndrome (CTS) is defined as a partial fracture typically involving a vital posterior tooth, presenting with a sudden and sharp pain during chewing or thermal stimuli. Diagnosing the condition is challenging, and the characteristic symptoms are often indistinguishable from other dental diseases, which is why CTS is often misdiagnosed as one of these other conditions [1–4]. If the teeth are not treated, the pulp may become inflamed, which can eventually lead to loss of the tooth.

While CTS has the potential to impact several posterior teeth, mandibular first molars are the ones that are most affected because of high occlusal forces [3]. This syndrome is difficult to diagnose as the crack lines might not be visible clinically, the symptoms change from one patient to another, and the accuracy of radiographic diagnosis is usually minimal. Consequently, clinicians are forced to decide on whether to go for conservative restorations, full-coverage crowns, endodontic therapy, or extraction without knowing the depth, orientation of the crack, and the exact pulpal status [2]. Such an indeterminate situation may have an impact on the prognosis and the level of patient satisfaction with the treatment [4].

The absence of a globally accepted diagnostic standard makes the identification of cracked teeth very challenging, and thus, the proper treatment is delayed most of the time. Usually, the diagnosis is done by seeing the response of the tooth to a restorative or endodontic procedure, i.e., it is not made at the first visit most of the time [1, 5]. Patients might present with vague or fluctuating symptoms, and cracks can behave unexpectedly, thus leading the clinician to over-treat or under-treat the condition [6, 7]. The existence of such a dilemma points out the necessity of evaluating and standardizing treatment approaches to arrive at a more accurate clinical decision which is guided by appropriate evidence. 

Systematic reviews focusing on the management of CTS are different in their range and methodology. Some of them limit their consideration only to posterior teeth, while the others differentiate between endodontically treated and untreated teeth, or between full-thickness and partial cracks [3]. They also differ in the assessment of various outcomes—failure rates, survival, or symptom relief [8]. Differences in the sets of inclusion and exclusion criteria used further separate the evidence, which makes it hard to compare the reviews. At present, there is no single synthesis for clinicians that would combine the results of all systematic reviews [9].

Umbrella reviews are significant as they consolidate evidence from diverse systematic reviews, thus offering a more comprehensive view of treatment effectiveness, contradictions, and areas lacking knowledge. Such reviews have served as a major tool for clinical decision-making and have been influential in the dental field, particularly in the development of guidelines in areas like periodontics and implantology [10, 11]. A great number of systematic reviews dealing with the treatment of CTS exist; however, there is no overview that combines their results. This hampers the establishment of consistent, evidence-based CTS management strategies.

This protocol specifies the procedures for an umbrella review concerning the safety and effectiveness of the current CTS treatments. The review, through the compilation of data from the existing systematic reviews, intends to provide clinicians with a more accurate and dependable basis for making treatment decisions and to recognize the areas where research is still insufficient [12–14]. 

### Objectives

With the ultimate goal of providing a better understanding of what the current body of evidence supports in healthcare settings, this umbrella review attempts to locate and carefully examine the systematic reviews that are currently available and have examined the effectiveness of different treatment approaches for cracked tooth syndrome (CTS). By analyzing the effectiveness of various therapies, evaluating the breadth and caliber of the available data, and pointing researchers in the direction of areas that need more research, this study seeks to help clinicians make decisions with more confidence. This review aims to provide clinicians with a consolidated understanding of the effectiveness of available CTS treatment options and to identify areas requiring further research [15].

### Research question

“What are the reported clinical success rates and complication profiles of various therapeutic interventions for Cracked Tooth Syndrome as synthesized from systematic reviews and meta-analyses?”

## Methods

### Protocol development

A multidisciplinary team comprising information specialists, research methodologists, and clinicians worked closely together to design the protocol. Our process for development of the protocol was transparent and comprehensive, and the PRISMA-P 2015 guidelines were followed. This included important elements like eligibility criteria, search strategy, data extraction, and plans for data synthesis. An additional file (Additional file 1) contains a comprehensive PRISMA-P checklist. The protocol was pre-registered with the PROSPERO database to improve transparency and prevent effort duplication. Many methodological aspects, especially those pertaining to determining bias risk and data synthesis techniques, were based on the Joanna Briggs Institute handbook [16, 17]. When combined, this methodology creates a precise and organized framework for carrying out an umbrella review that aims to be both methodologically sound and important in influencing future clinical judgments, research, and policy regarding cracked tooth syndrome (CTS).

### Review methodology

The increasing number of systematic reviews on treatment for the Cracked Tooth Syndrome (CTS) requires a higher-level synthesis to be able to bring out unambiguous evidence. A preliminary examination of existing systematic reviews suggested that an umbrella review would be the most appropriate approach to synthesize the varied and sometimes conflicting evidence [18]. This paper will synthesize data from higher-level evidence to perform the evaluation of different therapies in terms of their overall efficacy, safety, and consistency. The paper will strictly follow the PRISMA 2020 guidelines to ensure that the reporting is always complete and transparent [19].

AMSTAR 2, a well-known tool that evaluates both random and non-random systematic reviews, will be used to look at the methodological quality of the selected reviews [20]. For this review, we will use the Cochrane Handbook as well as the Joanna Briggs Institute Manual, both of which give a detailed recommendation and the overall framework and structure of an umbrella review, which will be used to conduct the review [9, 21].

A multidisciplinary team, along with information specialists, systematic review methodologists, and experienced endodontists, will be creating this project. Experts in methods and clinics will work together so that the review applies to practice and is executed soundly. To reduce bias and to preserve consistency, important elements such as the synthesis plan, quality assessment, and the literature search strategy will be very carefully planned. AMSTAR-2 will be used to appraise the methodological quality of each included review. Evidence duplication will be assessed using the Corrected Covered Area (CCA) method. This umbrella review seeks to give dentists a reliable summary of current CTS treatment studies. It will identify gaps in the literature as well, possibly influencing future studies [13].

### Inclusion criteria

#### Population (P)

This umbrella review would search for the systematic reviews of all the patients, regardless of age or gender, who have been diagnosed as having Cracked Tooth Syndrome (CTS) by clinical symptoms and the following clinical or radiographic findings: pain elicitation during biting, reaction towards hot or cold, and visible cracks. Reviews including populations with conditions unrelated to CTS (e.g., vertical root fractures, incomplete crown fractures not classified as CTS) will be excluded.

Intervention (I): it is expected that the systematic reviews that are considered eligible must present the treatment of CTS as their thematic focus. The treatments could be different and include both direct and indirect restorations. A few of them are: full crowns, onlays, splinting, endodontic therapy, extraction, and conservative or surgical treatments, which are the most common therapies to be applied for relieving symptoms or preserving tooth structure. Both single modality and multi-phase interventions will be considered. The dependency of the type of intervention and number of applications is not a crucial factor in the acceptability assessment of the study.

Comparator (C): eligible systematic reviews may include any comparator, such as alternative treatment approaches, different timings, or variations of the same intervention (e.g., immediate vs. delayed full-coverage restoration), placebo, or no treatment. Reviews with no comparison group will also be eligible.

Outcomes (O): in order to be eligible for inclusion, systematic reviews must document a minimum of one outcome that has clinical relevance, such as tooth survival, pain relief, restoration success, need for further treatment, functional outcomes, or patient-reported quality of life**.** Those reviews that only focus on diagnostic methods or biomechanical analysis, not followed by patient outcomes, will be rejected.

Type of studies: we will only consider systematic reviews and meta-analyses published in peer-reviewed journals. We will not include any primary research articles such as randomized controlled trials (RCTs), non-randomized clinical trials, cohort studies (prospective or retrospective), or case-control studies. Reviews without a clear methodology for study selection and data synthesis, as well as narrative reviews, will also be excluded. Only systematic reviews published in English will be included due to limitations in translation resources. This may introduce language bias; therefore, the number of non-English records excluded during screening will be documented and reported.

### Review characteristics

This section will summarize the key characteristics of the included systematic reviews, including publication year, number and type of primary studies, populations studied, interventions assessed, outcomes reported, and methodological features. These details will be extracted after study selection and presented in tables to provide an overview of the evidence base.

### Search strategy

#### Database search

Among the research protocols, a comprehensive and thorough search strategy will be executed to gather eligible systematic reviews and meta-analyses concerned with therapeutic measures for Cracked Tooth Syndrome (CTS). The search will be performed on five essential electronic databases: PubMed/MEDLINE, Embase, Scopus, Web of Science, and the Cochrane Database of Systematic Reviews (CDSR). Custom methods adjusted to the peculiarities of each database that combine both controlled vocabulary (e.g., Medical Subject Headings [MeSH]) and appropriate free-text queries will be used. Boolean operators (AND, OR) will be employed to allow for search sensitivity and specificity. Systematic reviews and meta-analyses filters will be imposed to narrow the search without any limitation on the publication date. In addition, the search will be confined to English-language papers only to guarantee comprehension and methodological consistency.

To broaden the range, the authors of this paper will go through the reference lists of all included reviews to find additional relevant publications. Experts in endodontics will also be asked for their opinion to confirm that the search for the reviews is complete. The search through the databases will be regularly updated while the review is being done to make sure the latest publication is included. This approach is in line with the Cochrane Handbook and PRISMA 2020 guidelines for umbrella reviews.

### Search terms

In order to create a complete research in this area, the use of controlled vocabulary (e.g., MeSH terms) and free-text keywords associated with Cracked Tooth Syndrome and its treatment will be combined to formulate the search. The search terms will identify the important issues such as the illness, the kind of intervention, and the possible effect of the intervention in the final decision of the treatment. The search terms will be adapted according to the indexing systems of each database. Boolean operators (AND, OR) will be applied to combine and optimize retrieval efficiency. The complete search strategies adapted for different databases can be found in Table 1.

**Table 1:** Complete search strategies adapted for different databases. Date of Search: June 1, 2025

Database	Strategy
PubMed	(“cracked tooth syndrome”[MeSH Terms] OR (“cracked”[All Fields] AND “tooth”[All Fields] AND “syndrome”[All Fields]) OR “cracked tooth syndrome”[All Fields] OR (“cracked”[All Fields] AND “tooth”[All Fields] AND “syndromes”[All Fields]) OR (“cracked tooth syndrome”[MeSH Terms] OR (“cracked”[All Fields] AND “tooth”[All Fields] AND “syndrome”[All Fields]) OR “cracked tooth syndrome”[All Fields] OR (“syndrome”[All Fields] AND “cracked”[All Fields] AND “tooth”[All Fields])) OR (“cracked tooth syndrome”[MeSH Terms] OR (“cracked”[All Fields] AND “tooth”[All Fields] AND “syndrome”[All Fields]) OR “cracked tooth syndrome”[All Fields] OR (“syndromes”[All Fields] AND “cracked”[All Fields] AND “tooth”[All Fields]))) AND (“Systematic Review”[All Fields] OR “Meta-analysis”[All Fields] OR “Review”[All Fields])
Scopus	(TITLE-ABS-KEY (“cracked tooth syndrome” OR “cracked tooth” OR “tooth fracture” OR “cuspal fracture”)) AND (TITLE-ABS-KEY (“systematic review” OR “meta-analysis”))
Embase	('cracked tooth syndrome'/exp OR 'cracked tooth syndrome' OR 'cracked tooth' OR 'tooth fracture'/exp OR 'tooth fracture' OR 'cuspal fracture') AND ('systematic review'/exp OR 'systematic review' OR 'meta-analysis'/exp OR 'meta-analysis')
Cochrane	(“cracked tooth” OR “cracked tooth syndrome” OR “tooth fracture” OR “incomplete tooth fracture” OR “vertical tooth fracture” OR “cracked molar” OR “split tooth”) AND (“systematic review” OR “meta-analysis” OR “review”) AND (“therapy” OR “treatment” OR “management” OR “intervention” OR “restoration” OR “endodontics” OR “crown” OR “bonded restoration” OR “occlusal adjustment” OR “extraction”)
Web of Science	(TS = (“cracked tooth syndrome” OR “cracked tooth” OR “fractured tooth” OR “incomplete tooth fracture”))AND(TS = (“treatment” OR “therapy” OR “management”))AND(TS = (“systematic review” OR “meta-analysis”))

### Study selection

To manage the references and remove duplicates, all records searched from the database will be imported into EndNote (Clarivate Analytics). After that, the citations that are left will be sent to Rayyan (Qatar Computing Research Institute), which is a tool for web-based screening that is meant to assist blinded as well as independent screening by the reviewers. Two reviewers will create separate assessments of titles and abstracts that are based on eligibility criteria that were pre-determined. Then, full-text articles of the relevant articles will be collected and checked for final inclusion. In case there is any disagreement during the process of screening, it will be solved through talk, or the third reviewer will be consulted. Any disagreements that continue and are not resolved through discussion between the two primary reviewers and the third adjudicating reviewer will ultimately be referred to a fourth senior reviewer for final decision. To demonstrate the selection process transparently and be able to track all the steps that are in line with the requirements of PRISMA 2020, a flow diagram will be used [19].

### Data extraction and management

To perform the data extraction, a structured, piloted extraction form will be used, which is designed specifically for this umbrella review (Additional file 2). To achieve comprehensiveness and consistency, two reviewers will independently extract relevant data from each overlapped systematic review [17, 20]. They will look for the year of publication, the aim of the review, the number and design of the primary studies, the characteristics of the study populations, the intervention and comparator details, the outcome measures, the synthesis methods, and the principal findings. Variables like heterogeneity (*I*^2^), effect sizes, and confidence intervals are going to be collected from studies, wherever there is such information, thus enabling comparative analysis among different reviews [22]. Any differences of opinion between the reviewers will be eliminated through a discussion, or, if it is still necessary, a decision given by a third reviewer will help to keep objectivity and reduce bias [21].

Extracted data will be handled in Microsoft Excel to provide full traceability of the entire process, allow for audits of the method, and ensure that the procedure of the review is carried out consistently [23]. If there are papers where the essential information is missing or is not clear, the authors of the original reviews will be contacted for a solution. This will help achieve a high level of clarity in the review, the possibility to reproduce it, and protection of the data throughout the process [17, 22].

Apart from the main variables that were taken from each systematic review, we are going to record the information of the funding sources, the primary study designs that are included in each review, the follow-up duration in the underlying studies, and the operational definitions of “success” or treatment effectiveness that are used across the reviews. These features will provide a background to the variability of the results and help to explain differences in the effectiveness that has been reported. Getting these additional variables will help us to compare the characteristics of the reviews more deeply and will also be a great help in determining how to synthesize the evidence from different sources that are not in agreement with each other. 

### Assessment of methodological quality

The methodological quality of the systematic reviews included in this umbrella review will be independently assessed by two reviewers using the AMSTAR-2 instrument [20]. AMSTAR-2 evaluates 16 methodological domains, allowing reviews to be rated as high, moderate, low, or critically low confidence. It examines factors such as the comprehensiveness of the literature search, the adequacy of risk-of-bias assessment, the appropriateness of statistical techniques, and the transparency of conflict-of-interest reporting [14]. Any disagreements between reviewers will be resolved through discussion or by consulting a third reviewer if necessary [20]. The results of this appraisal will support the interpretation of the evidence and will also be used for planned sensitivity analyses to explore how methodological quality may influence the robustness of the umbrella review’s conclusions [22]. This approach ensures that the synthesis is based on reliable and rigorously assessed evidence, in line with current guidelines for umbrella reviews [23, 24]. In addition to methodological quality appraisal, overlap of primary studies across systematic reviews is a recognized methodological challenge in umbrella reviews and may lead to biased or inflated conclusions if not explicitly addressed [25]. To quantify the extent of overlap, evidence duplication will be assessed using the Corrected Covered Area (CCA) method, which provides a validated quantitative measure of overlap among included reviews [26]. Furthermore, established methodological guidance will be followed to manage overlapping data and to ensure appropriate interpretation of findings when multiple systematic reviews include the same primary studies [27].

### Data synthesis

The data analysis will be done using a structured narrative strategy, which is the most suitable approach for umbrella reviews [9]. A detailed summary of each included review will be provided, including important variables such as populations, interventions, comparators, outcomes, the number of included primary studies, and principal findings, as well as obtained effect estimates and confidence intervals where applicable [28, 29]. Meta-analyses in incorporated reviews with analysis of pooled effect sizes, measures of heterogeneity (e.g., *I*^2^), and subgroup analyses will be evaluated to assess evidence uniformity and direction within the therapeutic interventions [21]. If we discover inconsistent or contradictory data, we will look closely at methodical variations such as inclusion criteria, risk of bias assessments, and quality of evidence grading to determine if they can provide insight into the disparities [24]. In order to guarantee transparency and accessibility, generated information will be presented both in a descriptive manner and in the form of tables, providing healthcare professionals and researchers with a clear and comprehensive overview of therapeutic strategies for CTS [20].

### Handling conflicting evidence

Apart from the narrative synthesis, the discrepancies or differences in results of the systematic reviews that have been included will be analyzed through a structured comparative approach. The differences will be examined in terms of the quality of the review (AMSTAR-2 scores), the timing of the reviews, the comprehensiveness of their search strategies, the way outcomes were defined, and any clinical or analytical variations. In case there are still inconsistencies that cannot be accounted for by these factors, they will be flagged and taken into consideration while deciding the overall direction and level of confidence in the evidence. 

### Sensitivity and subgroup analyses

Sensitivity analyses are planned to verify the stability of the results. It will comprise obtaining the results again after removal of the reviews rated as “critically low” or “low” quality on AMSTAR-2, and checking whether the overall conclusions differ when only moderate- or high-quality reviews are considered. Subgroup analyses will be performed based on clinically and methodologically relevant factors like type of intervention (restorative, full-coverage, endodontic, or extraction-based), endodontic status of the tooth, crack severity, and tooth location. Also, we will compare older and more recent systematic reviews to evaluate whether new evidence changes the conclusions. These analyses will be instrumental in determining the level of evidence stability and whether certain subgroups have more consistent treatment outcomes. 

### Assessment of reporting bias

In this umbrella review, reporting bias will be assessed indirectly at the level of the incorporated systematic reviews. We will use statistical approaches such as funnel plot asymmetry, Egger’s test, or Begg’s test [30, 31], to determine whether each incorporated review evaluated publication bias or small-study effects. Furthermore, we will record whether systematic reviews incorporated unpublished data, searched the grey literature, or used systematically registered protocols, as such variables may decrease the possibility of selective reporting [19, 32]. Reviews that did not assess reporting bias will be stated and interpreted accordingly. While direct assessment of reporting bias across primary studies is not possible throughout the umbrella review framework, our approach enables a transparent assessment of how thoroughly reporting biases have been tackled in the currently available body of evidence [28, 29]. This indirect assessment will allow for a deeper assessment of the overall confidence and dependability of the conclusion generated [17].

### Certainty of evidence

The implementation of the Grading of Recommendations, Assessment, Development and Evaluation (GRADE) framework in umbrella reviews is still a debated issue because GRADE was first created for primary studies and only later changed for systematic reviews [33, 34]. However, considering its organized and clear way of determining the certainty of the evidence, we will use GRADE in a pragmatic way in this umbrella review, which is in line with the current best practices for evidence synthesis at the review-of-reviews level. If the systematic reviews included in the study have already utilized GRADE, the corresponding evaluations will be taken and recorded as they are without any changes to maintain the consistency of the original assessments. 

If GRADE assessments have not been performed in systematic reviews, we will check whether there is enough information to stage a modified GRADE approach for umbrella reviews [9, 18]. The level of evidence will be evaluated through the usual GRADE domains—risk of bias, inconsistency, indirectness, imprecision, and publication bias—based on the aggregated results of the included reviews. The assessment will also consider the available pooled effect estimates, heterogeneity statistics (*I*^2^), risk-of-bias judgments from the primary studies, and indications of publication or reporting bias [35].

Where data are insufficient to perform a valid GRADE assessment, the lack of a certainty grading will be formally recorded, and the consequences for the interpretation of the results will be clearly stated [36]. The adoption of this combined approach makes sure that the methods used are transparent and, at the same time, considers the intrinsic limitations of the application of GRADE at the level of an umbrella review, thus improving the strength and clinical relevance of the evidence synthesis. 

### Ethics and dissemination

This umbrella review requires no ethical clearance because it will generate findings of already published systematic reviews without gathering new data from each participant [37]. The study will follow defined methodological guidelines for conducting umbrella reviews, facilitating accountability, accuracy, and uniformity throughout the entire process [17, 21]. This comprehensive analysis is intended to help guide the clinical setting, recognize discrepancies within the present evidence, and encourage potential recommendations in treatment strategies for CTS [28].

### Implications for clinical practice

The main findings of this umbrella review should be able to offer clinicians a broader, more complex summary of the treatment information for cracked tooth syndrome. Through aggregation of different systematic review studies, this umbrella review will make it clear what clinical treatments have the greatest standard of clinical success most of the time, which interventions have only limited effectiveness or lack effectiveness, and gaps in the evidence.

This synthesis will help clinicians to decide more effectively which treatment option—restorative, endodontic, or extraction-based—would be the best, especially in cases where the diagnosis is uncertain. Besides, it will facilitate the unification of decision-making pathways by clarifying prognostic factors, expected outcomes, and the comparative benefits of different treatment methods. 

Moreover, the results of this umbrella review might serve as a source of new guidelines for management of CTS by indicating the areas where there is clinical agreement and shedding light on those areas that require further high-quality studies. The review will also contribute to making the treatment outcome more predictable, and patient-centered care will be fostered in the management of cracked tooth syndrome. 

### Strengths and limitations of this study

Using thorough search techniques, two independent review methodologies, and organized data extraction, this umbrella review will combine evidence gathered from previous systematic reviews. Wherever possible, using GRADE will improve the outcome’s clinical validity and transparency. However, certain assessments may be limited by restrictions resulting from insufficient reporting, primary study overlap, and variations in the level of accuracy of included reviews. Throughout the evaluation and comprehension process, these factors will be openly discussed.

## Supplementary Information

Additional file 1. PRISMA-P 2015 Checklist for Protocol Submission.

Additional file 2. Sample Data Extraction Template.

## Data Availability

All data generated or analyzed during this study will be included in the published umbrella review and its supplementary files. No additional datasets were generated for this protocol.

## Abbreviations

AMSTAR-2: A Measurement Tool to Assess Systematic Reviews, Version 2
CCA: Corrected covered area
CDSR: Cochrane Database of Systematic Reviews
CI: Confidence interval
CTS: Cracked tooth syndrome
GRADE: Grading of Recommendations, Assessment, Development and Evaluation
I²: I-squared statistic (measure of heterogeneity)
JBI: Joanna Briggs Institute
MeSH: Medical Subject Headings
MEDLINE: Medical Literature Analysis and Retrieval System Online
PRISMA: Preferred Reporting Items for Systematic Reviews and Meta-Analyses
PRISMA-P: Preferred Reporting Items for Systematic Review and Meta-Analysis Protocols
PROSPERO: International Prospective Register of Systematic Reviews
RCT: Randomized controlled trial
RCTs: Randomized controlled trials
WOS: Web of Science
