# Supplementary material for: Saving the split: protocol for an umbrella review on therapeutic approaches for cracked tooth syndrome
Source: Syst Rev. 2026 Jan 7;15:41. doi: 10.1186/s13643-025-03048-y (PMC12870494; doi:10.1186/s13643-025-03048-y)
Supplement: Supplementary file 1 — Additional file 1. PRISMA-P 2015 Checklist for Protocol Submission. [file 13643_2025_3048_MOESM1_ESM.docx]

Addition File 1: PRISMA-P 2015 Checklist for Protocol Submission

Protocol Title: Saving the Split: Protocol for an Umbrella Review on Therapeutic Approaches for Cracked Tooth Syndrome

PROSPERO Registration ID: CRD420250648720

| Section/Topic | Item No. | Checklist Item | Reported on Page/Section |
| --- | --- | --- | --- |
| Title | 1a | Identify the report as a protocol of a systematic review | Title Page |
| Registration | 2 | If registered, provide the name of the registry and registration number | Page 1- Abstract |
| Authors | 3a | Provide names, affiliations, and contact information for all authors | Title Page |
| Contributions | 3b | Describe contributions of protocol authors and identify the guarantor | Stated in Title Page |
| Amendments | 4 | If the protocol represents an amendment, state this with explanation | No amendments have been made to this protocol at the time of submission. Any significant changes made after publication of this protocol will be documented in an additional file, clearly outlining the nature of the changes, the rationale, and the date they were implemented. These will also be updated in the PROSPERO registration accordingly. |
| Support | 5a | Indicate sources of financial or other support | Page 15: “Funding” |
| Sponsor | 5b | Provide name for the review funder and/or sponsor | Not applicable – Self-supported |
| Role of sponsor/funder | 5c | Describe roles of funder(s), sponsor(s), and/or institution(s) | Not applicable |
| Rationale | 6 | Describe the rationale for the review in context of what is known | Pages 3–5: Background |
| Objectives | 7 | Provide an explicit statement of the question(s) the review will address | Page 5 |
| Eligibility criteria | 8 | Specify the study characteristics and report characteristics to be used | Pages 7-8 |
| Information sources | 9 | Describe all intended information sources (databases, contact with authors) | Page 10-11, Database Search and Additional File 2 |
| Search strategy | 10 | Present draft search strategy to be used for at least one electronic database | Page 10 Additional File 2 |
| Study records | 11a | Describe how study records will be managed | Page 11- Study Selection |
| Selection process | 11b | State the process for selecting studies through each phase | Page 11- Study selection |
| Data collection process | 11c | Describe planned method of extracting data from reports | Page 11-12- Data Extraction and Management |
| Data items | 12 | List and define variables for which data will be sought | Page 11-12- Data Extraction and Management |
| Outcomes and prioritization | 13 | List and define all outcomes and provide rationale for prioritization | Page 8- Outcome |
| Risk of bias in studies | 14 | Describe anticipated methods for assessing risk of bias | Page 13-Assessment of Reporting Bias |
| Data synthesis | 15a | Describe criteria under which data will be quantitatively synthesized | Not applicable |
| Data synthesis | 15b | If appropriate, describe methods of handling data and combining results | Page 13- Data-synthesis |
| Data synthesis | 15c | Describe any proposed additional analyses (sensitivity, subgroup) | Page 13- Data-synthesis |
| Meta-bias(es) | 16 | Specify assessment of meta-bias(es) | Page 13- Data-synthesis |
| Confidence in cumulative evidence | 17 | Describe how the strength of the evidence will be assessed | Page 14-Certainty of Evidence |
