# Supplementary material for: Saving the split: protocol for an umbrella review on therapeutic approaches for cracked tooth syndrome
Source: Syst Rev. 2026 Jan 7;15:41. doi: 10.1186/s13643-025-03048-y (PMC12870494; doi:10.1186/s13643-025-03048-y)
Supplement: Supplementary file 2 — Additional file 2. Sample Data Extraction Template. [file 13643_2025_3048_MOESM2_ESM.docx]

# Additional File 2: Sample Data Extraction Template

This table will be used to extract data from included systematic reviews. It has been piloted and refined for use in this umbrella review.

| Review Author (Year) | Review Type | No. of Studies | Population | Intervention(s) | Comparator(s) | Outcomes | Key Findings | AMSTAR-2 Rating |
| --- | --- | --- | --- | --- | --- | --- | --- | --- |
